# Risk factors for the prognosis of pediatric medulloblastoma: a retrospective analysis of 40 cases

**DOI:** 10.6061/clinics/2017(12)12

**Published:** 2017-12

**Authors:** Jianzhong Yu, Rui Zhao, Wei Shi, Hao Li

**CLINICS 2017;72(5):294-304**

Retraction requested by the Editor of CLINICS in agreement with the authors

Retraction type: Redundant publication

Comments: The same article was published almost simultaneously in the journal TRANSLATIONAL NEUROSCIENCE AND CLINICS with a different title: “Factors affecting the prognosis of children with medulloblastoma: A single institution retrospective analysis of 40 cases” (http://www.tncjournal.com/EN/10.18679/CN11-6030/R.2017.003).

All articles submitted to CLINICS undergo strict quality control and are checked using iThenticate software before being submitted for peer review and before acceptance. The retracted article was checked on September 26, 2016, and February 24, 2017. Unfortunately, the article was published in TRANSLATIONAL NEUROSCIENCE AND CLINICS in May 2017, so there was no way of acknowledging the publication during the evaluation process. The authors submitted all required copyright transfer documents stating that the manuscript had not been submitted to any other journal.

The authors stated the following: *“It was an unintentional mistake caused by carelessness. After acceptance of our article in CLINICS, we attended a seminar. TRANSLATIONAL NEUROSCIENCE AND CLINICS indexed our paper after the seminar, and we knew nothing about this at that time. We misunderstood and thought that this would not influence the publication of our paper in CLINICS as TRANSLATIONAL NEUROSCIENCE AND CLINICS is a non-SCI journal, and the paper was indexed only internally.”*

